# Companies’ Responses to a Tax on Sugar-Sweetened Beverages: Implications for Research: Comment on "Understanding Marketing Responses to a Tax on Sugary Drinks: A Qualitative Interview Study in the United Kingdom, 2019"

**DOI:** 10.34172/ijhpm.2022.7619

**Published:** 2022-12-06

**Authors:** Judite Gonçalves

**Affiliations:** ^1^School of Public Health, Imperial College London, London, UK.; ^2^Nova School of Business and Economics, Universidade Nova de Lisboa, Lisbon, Portugal.

**Keywords:** Multi-tier SSB Taxes, Reformulation, Optimal Tax Design, Multidimensional Impacts, Confounding Factors

## Abstract

The paper by Forde et al, newly published in this journal, sheds light on how sugar-sweetened beverages (SSBs) companies may react to the introduction of a SSB tax. This commentary goes over the paper’s main findings and drafts implications for research on the impacts of SSB taxes. First and foremost, future research needs to assess the actual impacts of SSB taxes on companies’ actions, especially reformulation. Second, cross-country research, comparing large companies with similar beverage portfolios, could bring insights about the impacts of external factors, including different SSB taxes, on companies’ decisions. Third, SSB companies’ actions are potential confounders in empirical studies looking into the impacts of SSB taxes on prices, demand, or other outcomes. Researchers need to be aware of and discuss such aspects thoroughly in their studies, as the implications for the interpretation of results are evident.

 Increasingly, countries around the world are taxing sugar-sweetened beverages (SSBs). The goals are to improve population health by reducing sugar intake from soda and other sugary drinks, while raising government revenue that can be earmarked for health or other programs. On the demand side, SSB taxes are supposed to reduce sugar intake by decreasing SSB consumption, through higher SSB prices and raised awareness about the health harms of drinking SSB. On the supply side, SSB taxes can reduce sugar intake by incentivizing SSB companies to lower the sugar content in the beverages they produce (ie, reformulation). This incentive to reformulate only exists if the tax is multi-tiered, ie, if higher tax rates apply to beverages with greater sugar content, because companies can pay a lower tax rate by cutting back on added sugar. Some recent adopters of SSB taxes have opted for a multi-tier tax design (eg, Portugal, the United Kingdom). France, which had a single-tier SSB tax since 2012, introduced multiple sugar-dependent rates in 2018.^1^ Besides encouraging reformulation, the multi-tier design brings us closer to taxing grams of sugar, as opposed to taxing flatly litres of soda, which is preferable in terms of maximizing social welfare, because it is sugar that generates harm.^2,3^

 The literature on the impacts of SSB taxes is expanding quickly. A recent meta-analytic study pools the findings of 41 articles, covering 18 SSB taxes implemented all around the world, to estimate a tax pass-through to final consumer prices at 66%-98% (ie, price increases of 6.6%-9.8% for a 10%-equivalent SSB tax). Based on 33 articles, covering 16 SSB taxes, the same study estimates a 9%-20% reduction in SSB sales (95% confidence intervals).^4^ The heterogeneity in price and demand effects of SSB taxes is immense, across both jurisdictions and beverage types. Consumer responses also vary with household characteristics, such as socioeconomic status, although too few studies conduct subpopulation analyses to allow any robust conclusions. Crucially, we lack evidence on the (long-term) impacts of SSB taxes on the outcome of primary interest: population health. Moreover, we still know little about other effects of SSB taxes, such as their impacts on consumer awareness, consumption of substitution beverages and other food products (eg, sweet snacks), and SSB companies’ reactions, including reformulation and other product changes. For instance, we know that recipe reformulations took place in Portugal, South Africa, and the United Kingdom around the time of implementation of SSB taxes dependent on beverages’ sugar content.^5-7^ However, we do not know how much less sugar is contained in SSB as a direct result of the introduction of SSB taxes.

 The paper by Forde et al, newly published in this journal, fills a gap in the literature, by shedding light on how SSB companies may react to the introduction of a SSB tax.^8^ The context of the study is the United Kingdom, where a multi-tier SSB tax (“soft drinks industry levy”) was introduced in 2018. As motivated above, this type of SSB tax design entails specific incentives for companies, and albeit increasingly popular, it is still implemented in few countries, making this study particularly relevant and timely. Since April 2018, UK-based SSB manufacturers/importers pay a tax of £0.18 per litre on drinks with more than 50 and up to 80 grams of sugar per litre, and a tax of £0.24 per litre on drinks with more than 80 grams of sugar per litre. Another important feature of the UK’s SSB tax is that it was first announced two years before it was implemented, potentially providing plenty of time for companies to plan and adjust. Forde et al report the findings of 18 qualitative interviews with representatives from industry, academia, and civil society, conducted about one year after the implementation of the SSB tax. They condense those findings into a theoretical framework describing companies’ potential actions and decision-making processes in response to such a tax.

 The paper’s main findings are threefold. First, companies’ decision-making processes are continuous and iterative, and likely to be accelerated rather than prompted by the introduction of a tax. UK-based SSB companies were probably already taking actions before the tax was introduced (eg, reformulating their products, changing their messaging), whether in anticipation of the tax or in response to other factors, such as trends in consumer preferences. Second, attempting to maintain profits, companies may engage in a variety of marketing activities, including reformulation, development and acquisition of new products, changes in messaging, changes in product size, new public relations campaigns, changes in distribution and placement, and changes in packaging (listed roughly from most to less often mentioned by the interviewees). Third, companies are likely to react differently, with their actions depending on their specific internal (eg, beverage portfolio, brand strength) and external context (eg, consumer preferences, suppliers and retailers’ influence).

 These findings call for and can guide follow-up research, at the company-level, on the actual impacts of SSB taxes on companies’ marketing activities. The first main finding implies that researchers must “discount” existing trends in order to isolate the impacts of SSB taxes, which requires finding appropriate control groups.^3^ The second main finding suggests that reformulation, development, and acquisition of new products are especially relevant outcomes to analyse. The third main finding calls for distinguishing between the effects on groups of more or less similar companies, to account for differences in internal contexts. Moreover, it is worth noting that in several countries, the SSB market has two very large players with portfolios dominated by the same beverages (recall, eg, the two most popular cola-flavoured or lemon and lime-flavoured soda beverages, and which companies market them). Future research could perhaps compare large companies with similar beverage portfolios across countries, to draw insights about the impacts of external factors on their decisions (eg, different SSB taxes, competitor landscape, consumer preferences).

 Forde and colleagues’ findings may also help us understand the heterogeneity in price and demand effects of SSB taxes across countries and beverage types that is observed in the literature. For example, price overshooting in some countries or beverage groups could be associated with product reformulation or other marketing activities raising costs. Underwhelming reductions in demand for SSB could be a result of consumers perceiving new or reformulated drinks as healthier. Again, this calls for robust investigations of the impacts of multi-tier SSB taxes on reformulation, which could be the main channel through which SSB taxes might reduce sugar intake and improve population health. The previous examples also show that SSB companies’ actions are potential confounders in empirical studies looking into the (ex-post) impacts of SSB taxes on prices, demand, or other outcomes. Researchers need to be aware of and discuss such aspects thoroughly in their studies, as the implications for the interpretation of results are evident. For instance, researchers may search advertisements for new product releases or new recipes, or search supermarkets for changes in placement, packaging, or promotion activity. Similarly, studies simulating, ex-ante, the impacts of a new tax, or a change to an existing tax, could model different scenarios of companies’ responses.^9^ This would help policy makers to outmanoeuvre the industry’s counteractions and design the most effective policies. Importantly, tax pass-through to final consumer prices, as well as SSB demand, depend not only on SSB companies’ actions, but also on retailers’. Retailers’ marketing activities following the introduction of SSB taxes should also be the subject of future research.

 Beyond its implications for research, the study by Forde et al has significant policy implications as well. Possibly, the main one is the importance of a consistent and integrated public health strategy that acknowledges the influence of multiple external factors on companies’ actions. For instance, SSB companies are more likely to make their products healthier if this is what consumers demand. This suggests that promoting health literacy and raising awareness about the harms of SSB consumption via public health campaigns can enhance the effectiveness of a SSB tax.

 To conclude, the study alerts about an important dimension of the impacts of SSB taxes, which is how SSB companies may react. Companies’ reactions have the potential to undermine the goals of SSB taxes. Therefore, we need to understand them in order to imbed the right incentives in the (re)design of SSB taxes. The study raises many questions, but it also guides the search for the answers.

## Ethical issues

 Not applicable.

## Competing interests

 Author declares that she has no competing interests.

## Author’s contribution

 JG is the single author of the paper.
